# A clinical study of the efficacy of a single session of individual exercise for depressive patients, assessed by the change in saliva free cortisol level

**DOI:** 10.1186/1751-0759-7-18

**Published:** 2013-12-06

**Authors:** Megumi Ida, Itsurou Ida, Naoki Wada, Makoto Sohmiya, Masayuki Tazawa, Kenji Shirakura

**Affiliations:** 1 Department of Rehabilitation Medicine, Gunma University Graduate School of Medicine, 3-39-22, Showa-machi, Maebashi 371-8511, Japan; 2 Department of Neuropsychiatry, National Hospital Organization Takasaki General Medical Center, 36, Takamatsu-cho, Takasaki 370-0829, Japan; 3 Division of Rehabilitation Medicine, Gunma University Hospital, 3-39-15, Showa-machi, Maebashi 371-8511, Japan

**Keywords:** Depression, Physical exercise, Saliva free cortisol level, Augmentation therapy, Quality of life

## Abstract

**Background:**

The efficacy of physical exercise as an augmentation to pharmacotherapy with antidepressants for depressive patients has been documented. However, to clarify the effectiveness of exercise in the treatment of depression, it is necessary to distinguish the effect of the exercise itself from the effect of group dynamics. Furthermore, an objective measurement for estimation of the effect is needed. Previous reports adopted a series of group exercises as the exercise intervention and mainly psychometric instruments for the measurement of effectiveness. Therefore, this clinical study was done to examine the effectiveness of a single session of individual exercise on depressive symptoms by assessing the change in saliva free cortisol level, which reflects hypothalamic-pituitary-adrenocortical axis function that is disturbed in depressive patients.

**Method:**

Eighteen medicated patients, who met the DSM-IV-TR criteria for major depressive disorder, were examined for the change in saliva free cortisol levels and the change in subjective depressive symptoms before and after pedaling a bicycle ergometer for fifteen minutes. Within a month after the exercise session, participants conducted a non-exercise control session, which was sitting quietly at the same time of day as the exercise session.

**Results:**

Depressed patients who participated in this study were in remission or in mild depressive state. However, they suffered chronic depression and had disturbed quality of life. The saliva free cortisol level and subjective depressive symptoms significantly decreased after the exercise session. Moreover, the changes in these variables were significantly, positively correlated. On the other hand, although the subjective depressive symptoms improved in the control session, the saliva free cortisol level did not change.

**Conclusion:**

For the first time in depressive patients, we were able to show a decrease in the saliva free cortisol level due to physical exercise, accompanied by the improvement of subjective depressive symptoms. This identified a possible influence of exercise on the hypothalamic-pituitary-adrenal axis in depression.

These results suggest the utility of assessing the effect of physical exercise by saliva free cortisol level in depressive patients who suffer from bio-psycho-social disability.

## Background

In this clinical study, we aimed to clarify the efficacy of physical exercise on depressive symptoms by adopting a single session of individual exercise as the exercise format and by assessing the change in the saliva free cortisol level as an evaluation tool.

According to the International Classification of Diseases 10th Revision (ICD-10) [1], a person with depression suffers from a depressed mood, loss of interest and enjoyment, reduced energy leading to increased fatigability, and diminished activity. Depression often involves somatic symptoms such as disturbed sleep or appetite and weight loss, and it disturbs not only psychological but physical and social aspects of a person’s life. In addition, depression is essentially an episodic recurring disorder and often becomes chronic [2].

The first–line of treatment for depression is pharmacotherapy with antidepressants. However, the remission rate with selective serotonin reuptake inhibitors, which are currently the first-line medication, is only at most 60% [3]. Even after continuation and maintenance use of antidepressants after remission became common, the high rate of recurrence and chronicity of depressive episodes has not been resolved [4,5].

In addition, recent studies about psychosocial disability in depression disclosed that the disability, which is directly correlated with the severity of depression, chronically persisted, even in inter-episode or in remission periods [6,7]. Moreover, while depression affects individuals at any life stage, the incidence is the highest in middle age when a person must play important social and family roles [2]. In the Global Burden of Disease (GBD) Study, which is a large-scale survey of the health burden of all diseases that takes chronicity as well as mortality of diseases into consideration, in 2004 unipolar depressive disorders were ranked the third cause of the burden of all diseases, and they are estimated to be the first cause by 2030 for all ages [8].

An effective augmentative approach for use in addition to continuation and maintenance medication is strongly required for depressive patients.

The contribution of physical exercise to mental health has been reported since the 1980s. Morgan claimed that prevention of a sedentary lifestyle, which is common in modern life, was important for maintaining mental as well as physical health [9]. Additionally, it has been shown that physical activity can potentially prevent depression [10]. Several recent studies showed the efficacy of exercise in the treatment of depression as an augmentation for antidepressant treatment [11-13].

However, some problems elucidated in previous studies concerning the efficacy of exercise on depression remain to be clarified. Firstly, subjects attended a series of exercise sessions delivered within a group format. Therefore, it is difficult to distinguish the effect of the exercise itself from the effect of group dynamics. Secondly, the effect of exercise was measured mainly with psychometric instruments, such as self-administered testing (e.g., Beck Depression Inventory) or questionnaires by clinicians (e.g., Hamilton Rating Scale for Depression). To date, no objective measurements that reflect the pathophysiological mechanisms underlying depression have been used to evaluate the effectiveness of exercise.

To elucidate the latter problem, we focused on hypothalamic-pituitary-adrenocortical (HPA) axis dysfunction in depression, which is the most valid current neurobiological theory for explaining the pathophysiology of depression. The HPA axis consists of the interactions among the hypothalamus, the pituitary gland, and the adrenal cortex, and is a major part of the neuroendocrine system that controls reactions to stress [14]. The clinical manifestation of its dysfunction in depression includes basal hypercortisolemia [15], elevated cortisol secretion in the dexamethasone suppression test [16], and increased cortisol release in the combined dexamethasone suppression-corticotropine releasing hormone stimulation test [17,18].

Recently, the measurement of saliva free cortisol level has made it possible to explore the involvement of dysregulation of the HPA axis in the pathophysiology of depression [19]. Saliva free cortisol is a protein-unbound cortisol that exerts bioactivity of the hormone, which is well correlated with the serum cortisol level. Moreover, the determination of the saliva free cortisol level is a non-invasive method, which can avoid change in the concentration by stimulation of blood sampling in cases of serum cortisol determination [19].

For these reasons, by assessing the change in saliva free cortisol level before and after exercise, we felt that we would be able to objectively determine the efficacy of exercise on depressive symptoms and to clarify the biological mechanisms underlying the effect of exercise.

In this paper, we examined the efficacy of a single session of individual exercise on the depressive symptoms of depressive patients by assessing the change in the saliva free cortisol level.

To the best of our knowledge, this is the first report that used the saliva free cortisol level for the measurement of the effectiveness of physical exercise on depressive symptoms.

## Methods

### Participants

Between July 2008 and July 2009, 39 outpatients at the Department of Neuropsychiatry in the National Hospital Organization Takasaki General Medical Center who met the DSM-IV-TR criteria for major depressive disorder (MDD) [20] were asked to participate in this study. The purpose and procedures were explained by the patient’s psychiatrist. Exclusion criteria were comorbid conditions, including substance abuse, current medical conditions that might lead to global cognitive impairment or cerebrovascular risk factors, pregnancy that might cause alteration in adrenocortical activity, and specific medical contraindications for exercise.

Of the 39 who met the inclusion criteria, 18 agreed to participate.

### Study protocol

The eighteen participants were invited to an exercise session at our hospital. After one month, they were also invited to a control session that involved sitting quietly. If the participant had been given antidepressants before entry, the dosage was fixed for the duration of the study.

At both sessions, we assessed the change in the saliva free cortisol level of each participant and the change in subjective depressive symptom scores before and after the sessions in order to examine the effect of the exercise on the participants’ depressive symptoms.

All participants provided written informed consent after a full explanation of the study purpose and procedures. This study was conducted in accordance with the Declaration of Helsinki and was approved by the ethics council of the National Hospital Organization Takasaki General Medical Center.

### Exercise session

The exercise that we chose was pedaling a bicycle ergometer (Aerobike 2000U, COMBI, Japan) by oneself.

Before participants’ routine consultation, they were invited to practice pedaling the bicycle ergometer for 15 minutes at the same time of day (between 13:00 and 14:00). The exercise load was unified with the Borg Rating of Perceived Exertion Scale (Borg RPE Scale), which is very popular in health sciences for quantifying exertion. It has been translated into many languages, including Japanese [21]. The scale values range from 6 (exertion: very, very light) to 20 (very, very hard), which denote heart rates ranging from 60-200 beats per minute. The participants were instructed to regulate their speed of pedaling the bicycle ergometer within the range from 11 (exertion: fairly light) to 13 (somewhat hard) on the Borg RPE Scale. In addition, the participant’s heart rate was recorded to show the actual exercise load that was done and the change in autonomic nerve system function related to the exercise.

To insure that the discomfort caused by the unfamiliar environment would not affect the participant’s saliva free cortisol level, the bicycle ergometer was set up in the room next to the consulting room, and their doctor was with the participant during the exercise session.

### Control session

Within one month after the exercise session, the participants were asked to attend a control session. Of the 18 participants, three did not participate in the control session because they could not come to the hospital. Case 1 and 7 had reached complete remission and Case 8 had changed hospitals. As a result, the data of fifteen participants was available.

Also, before the routine consultation the participants were asked to sit quietly in a waiting room for 15 minutes. The control session took place at the same time of day (13:00-14:00) as the exercise session in order to exclude a change in the saliva free cortisol level based on the circadian rhythm.

### Assessment and outcome measures

#### Severity of depressive symptoms

Before the exercise session, the level of depressive severity was assessed with the 17-item Hamilton Rating Scale for Depression (HRSD-17) [22]. This is a widely used semi-structured interview that covers a range of affective, behavioral, and physical symptoms of depression. The scores range from 0 to 52, with a score of 0-7 considered to be within the normal range or clinical remission, a score of 8-19 indicating mild severity, and a score of 20 or higher indicating at least moderate severity. An experienced psychiatrist, the attending doctor of the participant, administered the HRSD-17.

#### Health related quality of life

Before the exercise session, we examined psychosocial disability by measuring the health related quality of life (HRQOL) of each participant with the Medical Outcome Study Short-Form 36-Item Health Survey (SF-36) [23]. SF-36 is based on multi-dimensional health concepts and consists of the subscales of eight dimensions and the two summary scales shown below that measure the full range of health states. It has proved to be a useful HRQOL survey for many patient populations, including depressive patients [24-26]. We adopted version 2.0 of the SF-36 Japanese edition (SF-36v2™), which has been validated for the Japanese population [27-29]. SF-36v2™ uses norm-based scoring, so that we can calculate each score compared to the national-norm of 50. A higher score than 50 indicates better HRQOL than the national standard.

The eight SF-36 subscales and two summary scales are:

● Physical functioning (PF): physical ability to do daily activities

● Physical role functioning (RP): physical ability to fulfill a daily role, such as work or housework

● Bodily pain (BP): perceived bodily pain

● Social functioning (SF): ability to socialize with family and friends

● General health (GH): subjective assessment of general health

● Vitality (VT): perceived physical energy

● Emotional role functioning (RE): ability to emotionally fulfill daily roles

● Mental health (MH): feelings of depression and nervousness, or relaxation and fun. From the aggregation of these subscales, two distinct higher-level summary scores are available.

● Physical component summary (PCS): general physical health state

● Mental component summary (MCS): general mental health state

#### Participants’ preferences for physical exercise and their physical activity habits

Before the exercise session, the participants’ preferences for physical exercise, their daily exercise habits, and their reasons for exercising were asked via written inquiries and interviews. The questions were a) “Do you like exercise?” with answers “yes”, “no” or “uncertain”, b) “What kind of exercise do you actually do?” and c) “Why do you exercise?”.

#### Saliva free cortisol level

A saliva sample was collected from each participant three times during the exercise and control sessions: just before the session, immediately after the session, and 10 minutes later.

The reason for selecting 10 minutes after as the end point of saliva sampling is that it took 10 minutes for the heart rate at the end of the exercise period to return to the pre-exercise rate in our preliminary examination (Data not shown). At each saliva sampling, the participant was asked to bite a small cotton roll for 90 seconds, after which the cotton roll, including the participant’s saliva, was placed into a container (Salivette™, SARSTEDT AG & Co., Germany) designed to be able to remove the saliva from the cotton by centrifugation. During both sessions, the participant was instructed to refrain from gargling in order to prevent a change in the saliva free cortisol level.

The container was centrifuged at 1,000 g for 2 minutes and stored at -20°C until biochemical analysis. The saliva free cortisol level was measured by radioimmunoassay at the SRL Corporation (Tokyo, Japan) using a radioimmunoassay kit with ^125^I, (GammaCoat™ Cortisol, DiaSorin, Inc., Stillwater, MN). The saliva free cortisol level detection range of this kit is 0.06–9990 μg/dL.

#### Subjective depressive symptom score

To evaluate the change in subjective depressive symptoms before and immediately after the exercise and control sessions, eight depressive symptoms were self-rated.

Six of these symptoms, which we expected to be improved by a single session of exercise and that were easy for the participants to understand were excerpted from HRSD-17, a widely used semi-structured interview for the diagnosis of the severity of depression that has proven validity as a psychometric measure [22]. Moreover, it has been used as the outcome measure for assessing the effect of exercise in depressive patients [11-13]. The six symptoms we selected are depressed mood, disability of concentration (retardation is included in HRSD-17), agitation, anxiety, fatiguability (included in the somatic symptoms of HRSD-17), and hypochondriasis. In addition, we added two symptoms to the subjective depressive symptom score for this study. Perceived stress level and confidence about self-ability were not included in HRSD-17, but were expected to be improved by a single session of exercise.

Each symptom was scored on a four-point scale, ranging from 0 to 3. A score of 0 represents the absence of that subjective symptom, and the scores of 1, 2, and 3 indicate mild, moderate, or severe symptom respectively, except for confidence about self-ability. For this symptom, a higher score represents the lower confidence; the score was reversed when calculating the total subjective depressive symptom score.

### Statistical analysis

All statistical analyses were performed using R version 2.9.0, a free software program for statistical computing [30]. The changes in the saliva free cortisol levels before, immediately after, and 10 minutes after the exercise and control sessions were evaluated with the Friedman-rank-sum-test. The changes in the subjective depressive symptom scores before and immediately after the sessions were evaluated with the Wilcoxon-signed-rank-test. These tests were adopted because the results of the Shapiro-Wilk test for saliva free cortisol level and the subjective depressive symptom scores before the exercise session suggested that these two variables were non-parametric.

The correlation between the changes in saliva free cortisol level and the changes in subjective depressive symptom scores before and immediately after the sessions were evaluated with the Spearman's-rank-correlation-coefficient. The correlation between the pre-exercise saliva free cortisol level and, HRSD-17 scores and SF-36v2™ scores were evaluated likewise.

## Results

### Participant characteristics

Demographic and clinical characteristics of the eighteen participants at entry to the study are shown in Table 1.

**Table 1:** Demographic and clinical characteristics of all eighteen participants

**Case No.**	**Age (years)**	**Sex**	**Duration of illness (months)**	**Duration of medication (months)**	**Duration of current depressive episode (months)**	**Number of previous depressive episodes**	**Dose of antidepressants (mg/day)**	**HRSD-17 score(0-54)**	**SF-36: PCS**	**SF-36: MCS**
1	31	M	45	44	3	4	SULP (150) AMI (150)	14	−7.2	34.4
2	27	F	27	27	16	1	SER (150)	8	38.6	46.7
3	55	F	9	9	9	2	TRZ (75) MIA (10)	10	35.3	56.0
4	56	F	103	103	6	4	AMI (150)	14	20.2	26.8
5	28	F	14	14	14	0	SER (100)	14	36.6	39.4
6	37	M	20	20	20	1	SER (100)	13	5.1	39.3
7	61	F	105	105	6	3	SER (125)	6	49.6	56.9
8	38	M	62	62	3	0	AMI (150)	14	39.7	26.5
9	32	F	42	42	12	1	AMI (25)	8	18.9	47.3
10	44	F	316	316	14	6	SULP (100)	7	25.2	51.0
11	53	M	7	7	7	0	SER (75)	6	23.5	52.3
12	37	M	84	84	1.5	3	MIL (150)	22	28.2	25.3
13	56	M	10	5	6	0	PAROX (40)	9	NA	NA
14	53	F	9	9	10	0	SER (75)	5	49.3	64.9
15	68	F	18	18	8	1	MIL (100)	3	51.5	64.3
16	61	F	204	144	11	1	MIL (50)	6	35.5	40.4
17	31	M	12	12	12	0	MIL (100)	8	48.4	39.4
18	58	F	12	12	12	0	AMI (100)	3	40.3	36.7
Mean	45.9	M = 7	61.1	57.4	9.5	1.5		9.4	31.7	44.0
SD	13.3	F = 11	81.5	76.6	4.9	1.8		4.9	16.2	12.4

### Severity of depression

Each participant’s HRSD-17 sore is shown in Table 1. Seven participants were in clinical remission (HRSD-17≦7), ten suffered from mild depression (8≦HRSD-17≦19), and one suffered from moderate depression (HRSD-17≧20). All participants were taking therapeutic doses of antidepressants for the purpose of acute phase treatment, continuation, or maintenance treatment for preventing recurrence.

However, the mean duration of illness was 61.1 ± 81.5 months, which indicated long-term depression. Moreover, as shown by the number of previous depressive episodes, not a few of them had repeated recurrence, even though they were under long-term medication.

Furthermore, the participants’ mean total HRSD-17 score before the control session was 8.3 ± 6.8, a non significant difference from the mean HRSD-17 score before the exercise session (9.5 ± 4.9).

### HRQOL

PCS and MCS scores are shown in Table 1. Moreover, the mean SF-36v2™ eight subscale scores and two summary scale scores of all eighteen participants are shown in Table 2.

**Table 2:** **The mean SF-36v2**^**TM **^**eight subscale scores and two summary scale scores of all eighteen participants**

**SF-36 scales**	**Mean score**	**SD**
Physical functioning (PF)	40.4	15.2
Physical role functioning (RP)	31.2	15.7
Bodily Pain (BP)	42.7	14.9
General health (GH)	41.5	10.8
Vitality (VT)	42.7	15.5
Social functioning (SF)	33.8	16.3
Emotional role functioning (RE)	32.1	16.2
Mental health (MH)	42.6	13.3
Physical component summary (PCS)	31.7	16.2
Mental component summary (MCS)	44.0	12.4

All SF-36v2™ subscale scores and summary scale scores were lower than the national-norm of 50. Especially, RP and RE scores, which indicate how a person can fulfill daily roles like work and household chores, physically and mentally, and scores of SF, which indicate how a person socializes with family members, friends and companions, were remarkably low. In addition, the PCS score, which represents general physical health, was lower than the MCS, which represents general mental health.

### Correlation between severity of depression and HRQOL

The SF-36v2™ subscale scores, except for PF and BP and two summary scores, of all participants were significantly, negatively correlated with their total HRSD-17 scores.

RP, MH and MCS scores were strongly correlated to total HRSD-17 scores, with correlation coefficients of -0.79, -0.78, and -0.72 (p < 0.01), respectively. RE, VT, SF, PCS and GH were moderately correlated to total HRSD-17 scores, with correlation coefficients of -0.68, -0.64, -0.58, -0.55 and -0.52 (p < 0.05), respectively. The severity of depressive symptoms corresponded with the decline in HRQOL scores.

### Participants’ preferences for physical exercise and their physical activity habits

Of the 18 participants, 17 reported to like exercise and actually did some kind of routine exercise. The exercises that most of the participants participated in were taking walks near their house (12 participants) and cycling (2 participants). Their reasons for exercising were as follows, maintaining or promoting health, changing their mood, losing weight, prevention of aging, and getting out of their house. Some participants exercised because of their family’s recommendation.

Our participants performed exercises as a favorite pastime, but they also exercised in order to improve their state of health and quality of life.

Some participants realized an improvement in their mood during and after exercise, but some answered that they still could not escape from unnecessary thoughts during exercise.

### Smoking status

None of the participants were habitual smokers, and none smoked during the study.

### Results of the sessions

#### Exercise load

During the exercise session, the mean Borg RPE Scale score was 11.7 ± 1.1. Thus, the exertion during the exercise session was fairly light and within the range that the participants were instructed to perform (Borg RPE Scale: 11-13).

The mean heart rates before, immediately after, and 10 minutes after the exercise session were 83.4 ± 18.6, 112.3 ± 9.4, and 87.5 ± 17.1 beats per minute, respectively. Importantly, the heart rate returned to the pre-exercise value within ten minutes. Also, the value just after the exercise session corresponded with the Borg RPE Scale score during the exercise. This shows that the participants’ autonomic nerve function as monitored by their heart rates had returned to the pre-exercise state ten minutes after the exercise session.

#### Correlation between the pre-exercise saliva free cortisol level and HRSD-17, SF-36v2^TM^ scores

The pre-exercise saliva free cortisol level was significantly, positively correlated with the total HRSD-17 scores (r = 0.54, p < 0.05). In addition, the pre-exercise saliva free cortisol level was negatively correlated with two SF-36v2™ subscale scores, RP and VT, with correlation coefficients of -0.52 and -0.50 (p < 0.05). The pre-exercise saliva free cortisol level corresponded to the severity of depressive symptoms and a decline in the physical aspect of HRQOL.

#### Saliva free cortisol level change

**Figure 1:**
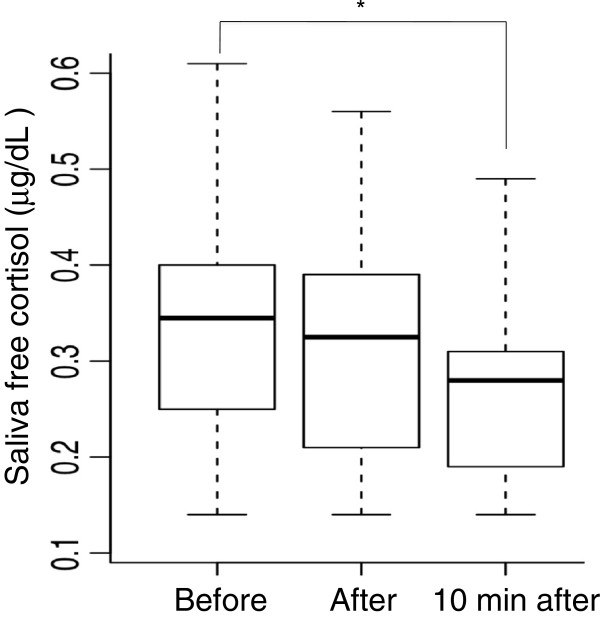
**Boxplot of time course change in the saliva free cortisol levels of the 18 participants during the exercise session.** Each box includes 50% of the cases (between percentils 25 and 75). The black line inside represents the median and the whiskers show extreme cases of individual variables. *p < 0.05 as compared to value before the exercise.

Figure 1 shows the change in the saliva free cortisol level of all eighteen participants during the exercise session. The saliva free cortisol level was decreased significantly before, immediately after, and ten minutes after the exercise session (Friedman rank sum test: χ^2^ = 11.303, df =2, p < 0.001). In addition, there was a significant difference in the saliva free cortisol levels before and 10 minutes after the exercise (Wilcoxon signed rank test: p < 0.05).

**Figure 2:**
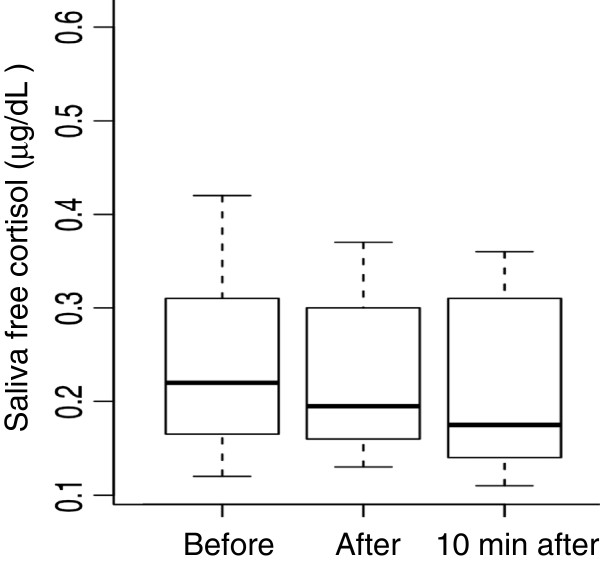
**Boxplot of time course change in the saliva free cortisol levels of 15 participants during the control session.** Each box includes 50% of the cases (between percentils 25 and 75). The black line inside represents the median and the whiskers show extreme cases of individual variables.

The changes in saliva free cortisol levels in the control session are shown in Figure 2. In contrast with the exercise session, the overall saliva free cortisol levels did not change during the session. Also, there were no significant differences in the saliva free cortisol level in comparison of the three testing points.

#### Change in subjective depressive symptom scores

**Figure 3:**
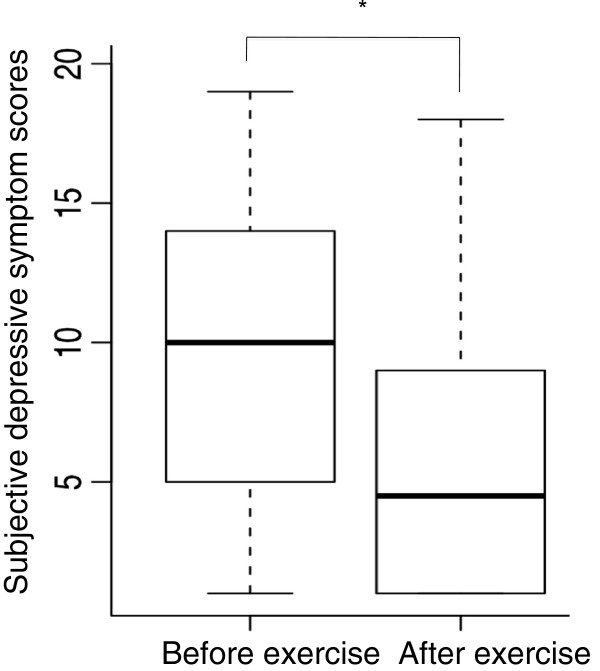
**Box plot of change in the subjective depressive symptom scores of the 18 participants before and after the exercise session.** Each box includes 50% of the cases (between percentils 25 and 75). The black line inside represents the median and the whiskers show extreme cases of individual variables. *p < 0.01 as compared to the value before the exercise.

**Figure 4:**
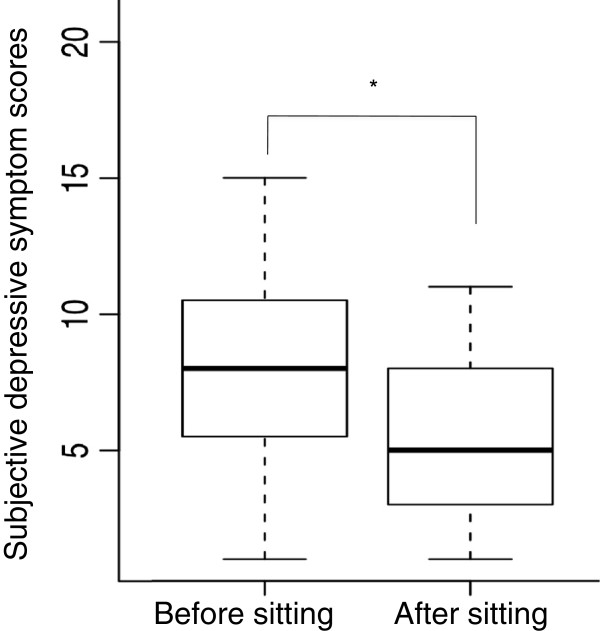
**Boxplot of change in the subjective depressive symptom scores of 15 participants before and after the control session.** Each box includes 50% of the cases (between percentils 25 and 75). The black line inside represents the median and the whiskers show extreme cases of individual variables. *p < 0.01 as compared to the value before the control session.

The changes in subjective depressive symptom scores before and after both the exercise and control sessions are shown in Figure 3 and Figure 4. Scores shown in the figures are the total of the eight subjective depressive symptom scores as perceived by the participants. The subjective depressive symptom scores were significantly decreased in both sessions (p < 0.01).

#### The relationship between the changes in the saliva free cortisol level and the changes in the subjective depressive symptom scores

**Figure 5:**
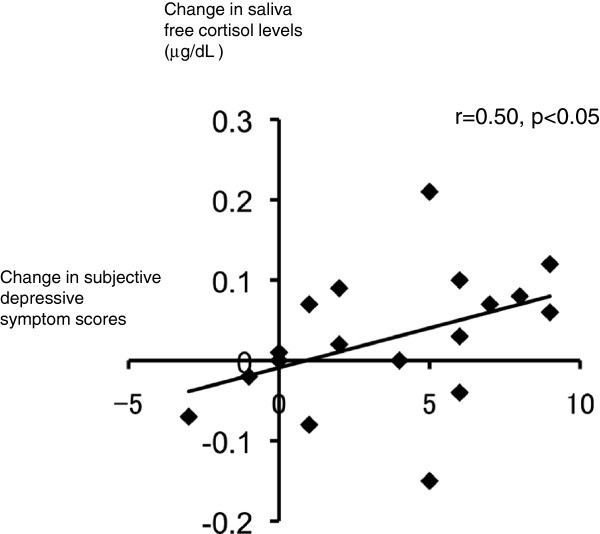
Correlation between the changes in the saliva free cortisol levels of the 18 participants before and immediately after the exercise session (scores of the pre-exercise session minus scores immediately after the exercise session).

As shown in Figure 5, the changes in the saliva free cortisol levels of all eighteen participants pre and immediately after the exercise session (i.e. scores of pre-exercise session minus scores immediately after the exercise session) were significantly correlated to the changes in the subjective depressive symptom scores (r = 0.50, p < 0.05). However, in the control session, no significant correlation was shown between the changes in saliva free cortisol levels and the changes in subjective depressive symptom scores.

## Discussion

### The relationship between our participants’ characteristics and the possibility of exercise as an augmentation therapy for depression

Almost all of our participants were in remission or in mild depressive state treated with antidepressants. However, their duration of illness was long and their HRQOL, which was directly correlated with the severity of depression, remained lower than the national-norm.

HRQOL, which is a reflection of the psychosocial disabilities of depressive patients, is a useful outcome measure for depression and an important target for improvement through treatment [31]. In line with our findings, previous reports demonstrated that depression has a profound impact on patient’s HRQOL [32,33].

Furthermore, recent studies exploring the HRQOL of depressive patients disclosed disturbances in the physical as well as the mental aspects of HRQOL [7,34]. For our participants’ HRQOL, the physical aspects, RP, SF, and PCS were remarkably low.

This fact deserves attention in considering the efficacy of exercise on depressive symptoms because it goes without saying that exercise acts on physical aspects of individuals. Moreover, our participants themselves chose to exercises daily in order to improve their health and quality of life.

For these reasons, exercise would naturally be acceptable for depressive patients and, moreover, the exercise is a potentially effective augmentation for antidepressant therapy, improving HRQOL by directly acting on physical ability, which is disturbed in depressive patients.

### The relationship between the efficacy of exercise and the change in saliva free cortisol level of depressive patients

As mentioned previously, the saliva free cortisol level is a biological indicator that reflects HPA axis dysfunction involved in the pathophysiology of depression. In fact, in our study, the saliva free cortisol level before the exercise session corresponded with the severity of depression and decline in the physical aspects of HRQOL (RP and VT). By adopting this indicator as the measurement of the effectiveness of physical exercise on depressive symptoms, we feel that it is possible to objectively examine the effect of exercise and, moreover, to explore whether or not exercise influences the HPA axis function of depressive patients.

The most important finding in our study is that the saliva free cortisol level is significantly decreased by a single session of individual exercise that does not include group dynamics. Moreover, the decrease in the saliva free cortisol level was correlated with a decrease in the subjective depressive symptom scores. This correlation was statistically significant.

On the other hand, although subjective depressive symptom scores improved in the control session, the saliva free cortisol level did not change. Our data show that the efficacy of physical exercise itself on depressive symptoms is not only a subjective impression, but an objective fact, as shown by the change in the saliva free cortisol level.

To our best knowledge, this is the first paper to clarify the relationship between the efficacy of physical exercise on depression and the change in saliva free cortical level, reflecting the HPA axis function disturbance of depressive patients. The following facts support these findings. To date, there are only two studies comparing cortisol response to physical exercise by depressive patients with that of healthy controls. Kiive et al. [35] reported no difference in serum cortisol response to intensive physical exercise by a group with depression and healthy controls. Moreover, Krogh et al. [36] observed more blunt cortisol response to exercise in depressed participants than in healthy controls. In previous studies, physical exercise was adopted as a stressor, not as a treatment. Moreover, serum, not saliva free cortisol level was measured. Thus, there have been no reports concerning the relationship between the therapeutic effect of physical exercise and the change in saliva free cortisol level.

To what extent do our present findings explain the influence of exercise on HPA axis function? In pharmacotherapy with antidepressants, numerous researchers have used the saliva free cortisol level to examine the association between their anti-depressive effect and the influence on the HPA axis function of depressive patients. These studies have proven that several antidepressants, including TCA [37], SSRI [38], SNRI and NaSSa [39] improved depressive symptoms, with a reduction of the saliva free cortisol level. These reports suggested that the efficacy of antidepressants is exerted via the recovery of the HPA axis dysfunction involved in the pathophysiology underlying depression.

The present results show that the anti-depressive efficacy of physical exercise itself has a similar mechanism to that of antidepressants, because exercise also induces a reduction of the saliva free cortisol level. However, to confirm this, further examination will be necessary to prove that the reduction of saliva free cortisol level of drug-free depressive patients is induced by exercise. Furthermore, if healthy subjects show a significantly lower basal saliva free cortisol level and more blunt response to physical exercise as compared with depressive patients, we could more strongly confirm the hypothesis. To that end, further investigation is needed.

### Limitations

Our present study has several limitations.

Firstly, the sample size was small and all of the participants were medicated during the study period. Further study with a larger number of participants and that includes drug-free patients will be necessary.

Secondly, the subjective depressive symptom scores, which evaluate the change in subjective depressive symptoms during intervention, was not sufficiently validated and must be confirmed in a future study with a larger sample size.

Thirdly, the interventions were not conducted with a counterbalanced design to control for order effect.

Fourthly, we did not clarify the effect of repetitive exercise. Therefore, we did not examine the long-term effect of physical exercise on the change of saliva free cortisol level.

We must overcome the issues above in future study to establish that physical exercise is useful for augmenting antidepressant therapy for depressive patients, and to clarify the anti-depressive effect of physical exercise by assessing the change in saliva free cortisol level.

## Conclusion

### The value of assessing the effect of exercise by the saliva free cortisol level of depressive patients

In this clinical study, we documented for the first time a decrease in the saliva free cortisol level due to exercise itself, and that the decrease was accompanied by an improvement in the subjective depressive symptoms of depressive patients. Although the number of participants was small, the efficacy of a single session of independent exercise by depressive patients was objectively confirmed. Moreover, exercise was shown to be a naturally acceptable intervention for depressive patients, as shown by our participants’ reported preferences for exercise and their daily exercise habits.

Our results suggest the utility of the saliva free cortisol level for assessing the effect of exercise by depressive patients who suffer from bio-psycho-social disability.

## Abbreviations

AMI: Amitriptyline; Borg RPE Scale: Borg rating of perceived exertion scale; BP: Bodily pain; DSM-IV-TR: The Text revision of the diagnostic and statistical manual of mental disorders 4th edition; GBD: The global burden of disease; GH: General health; HPA axis: Hypothalamic-pituitary-adrenocortical axis; HRSD-17: The 17-item Hamilton rating scale for depression; HRQOL: Health related quality of life; ICD-10: The International classification of diseases 10th revision; MCS: SF-36v2™ mental component summary; MH: Mental health; MIA: Mianserin; MIL: Milnacipran; NaSSa: Noradrenergic and specific serotonin antidepressants; PAROX: Paroxetine; PCS: SF-36v2™ physical component summary; PF: Physical functioning; RE: Emotional role functioning; RP: Physical role functioning; SER: Sertraline; SF: Social functioning; SF-36: Medical outcome study short-form 36-item health survey; SF-36v2™: The version 2.0 of SF-36 Japanese edition; SNRI: Serotonin norepinephrine reuptake inhibitor; SSRI: Selective serotonin reuptake inhibitor; SULP: Sulpiride; TCA: Tricyclic antidepressant; TRZ: Trazodone; VT: Vitality.

## Competing interests

The authors declare that they have no competing interests.

## Authors’ contributions

MI designed the study protocol, conducted the sessions, analyzed the data and drafted the manuscript. II treated the patients, conducted the sessions and drafted the manuscript. NW, MS and MT advised on data analysis. KS looked over the study. All authors read and approved the final manuscript.
